# “It’s like she’s talking about me” — Exploring the value and potential impact of a YouTube film presenting a qualitative evidence synthesis about chronic pain: An analysis of online comments

**DOI:** 10.1080/24740527.2020.1785853

**Published:** 2020-09-24

**Authors:** Francine Toye, Kate Seers, Karen Barker

**Affiliations:** a Physiotherapy Research Unit, Oxford University Hospitals NHS Foundation Trust, Oxford, UK; b Nuffield Department of Orthopaedics, Rheumatology and Musculoskeletal Sciences, University of Oxford, Oxford, UK; c Warwick Research in Nursing, Warwick Medical School, University of Warwick, Coventry, UK

**Keywords:** qualitative research, chronic pain, arts-based health research, film, impact, YouTube, internet, qualitative evidence synthesis

## Abstract

**Background:**

There is very limited research exploring the value and impact of qualitative research in chronic pain despite the large volume of research.

**Aims:**

The aim of this study was to find out whether viewers’ comments in response to a YouTube film, portraying findings from a qualitative evidence synthesis about living with pain, revealed any potential value or impact to viewers.

**Methods:**

We collected online data posted in response to the film *Struggling to Be Me*. We used themes from a large review of qualitative research as an a priori analytic framework. We used inductive thematic analysis to distil the essence of data that did not fit this framework. A thematic analysis of online comments to evaluate the impact of an arts-based health research film on people living with chronic pain is presented.

**Results:**

We developed two inductive themes that explored the value and potential impact of watching the film online: (1) It has given voice to our suffering and (2) it makes me feel that I am not alone. Two subthemes added insight to the a priori framework: First, *I have had enough of me* added insight to the theme *my life is impoverished and confined*; second, *I am treated like a criminal because I take opioids* added insight to the theme *lost personal credibility*.

**Conclusions:**

Our findings indicate that watching the YouTube film has potential value and impact, giving voice to suffering and making people feel that they are not alone. There are specific ethical challenges relating to internet-mediated research.

## RÉSUMÉ

**Contexte**: Malgré le grand volume de recherche existante, il existe très peu d’études portant sur la valeur et l’impact de la recherche qualitative en matière de douleur chronique.

**Objectifs**: Le but de cette étude était de savoir si les commentaires de personnes ayant visionné un film sur YouTube, qui dépeignait les résultats d’une synthèse des données probantes qualitatives sur la vie avec la douleur, révélaient que celui-ci pouvait avoir une valeur pour les personnes l’ayant visionné ou s’il pouvait avoir un impact pour elles.

**Méthodes**: Nous avons collecté des données en ligne publiées en réponse au film *Struggling to Be Me*. Comme cadre analytique préalable, nous avons utilisé des thèmes provenant d’une vaste revue de la recherche qualitative. Nous avons utilisé l’analyse thématique inductive pour distiller l’essence des données qui ne correspondaient pas à ce cadre. Une analyse thématique des commentaires en ligne pour évaluer l’impact d’un film artistique sur la recherche en santé portant sur les personnes vivant avec la douleur chronique est présentée.

**Résultats**: Nous avons élaboré deux thèmes inductifs qui ont examiné la valeur et l’impact potentiel du visionnement du film en ligne : (1) Il a donné la parole à nos souffrances et (2) il me fait sentir que je ne suis pas seul. Deux sous-thèmes ont ajouté de la profondeur au cadre préalable: Premièrement, j’en ai assez de moi a ajouté de la profondeur au thème ma vie est appauvrie et confinée; deuxièmement, je suis traité comme un criminel parce que je prends des opioïdes a ajouté de la profondeur au thème de la perte de crédibilité personnelle.

**Conclusions**: Nos résultats indiquent que regarder le film sur YouTube a une valeur et un impact potentiels, en donnant la parole à la souffrance et en faisant sentir aux gens qu’ils ne sont pas seuls. La recherche par Internet soulève des défis éthiques particuliers.

## Background

There is very limited research exploring the value and impact of qualitative research in the field of chronic pain, despite the large volume of research in this area.^1^ In 2012 the UK National Institute of Health Research (Health Services & Delivery Research) funded a study to synthesize findings from qualitative research about the experience of living with chronic pain,^2^ along with a film that portrayed the findings.^3^ This qualitative evidence synthesis (QES) was drawn from studies of more than 1000 adults with chronic pain from around the world, including the United States, Canada, the United Kingdom, Australia, New Zealand, Switzerland, Finland, The Netherlands, Norway, and Sweden. Narrative examples to illustrate each QES finding were selected and crafted into a script, which was performed by an actor. The film, *Struggling to Be Me with Chronic Pain*,^3^ described the erosion of a person’s sense of self, the struggle to negotiate health care, and the struggle to gain legitimacy in the face of invisible pain. It was posted on YouTube in 2013 and has now been accessed by more than 66,000 viewers.

Arts-based health research such as film, art, or theater can be a useful way to facilitate dissemination and can increase the impact of research findings. There are several reasons why arts-based health research can provide a powerful stimulus for change: it can make research accessible without oversimplifying, it can create a safe place for dialogue about challenging issues, it can trigger emotional engagement to facilitate change, and it can also challenge *ivory tower* representations of research.^4–6^ Research indicates that when health care professionals watched and discussed this film in an educational setting, it encouraged them to “see” the patient and that this facilitated a subtle change in perspective.^6^ We aimed to find out whether viewers’ comments in response to the film, posted on YouTube, revealed potential value or impact for people living with pain who watched the film.

## Method

### Methodological Approach

We provide a thematic analysis of online comments to evaluate the impact of arts-based health research film on people living with chronic pain.

### Data Collection

We copied all comments and responses to comments posted about the YouTube film *Struggling to Be Me with Chronic Pain* from inception until the end of September 2019. Confidentiality is challenging when using internet narratives. Even though the data were collected from the public domain, we took measures to reduce potential harm from threats to confidentiality. We took several measures to ensure confidentiality: (1) we removed overtly identifying information, such as geographical location or names; (2) we deconstructed narratives and reconstructed sections into a composite narrative; (3) we rephrased the data if we felt that it would allow a reader to return to the source data, while retaining the original meaning; (4) we cut, pasted, and searched for each individual sentence on google and YouTube to ensure that our reconstructed narrative could not be used to link narrative to its source. We uploaded the anonymized data to NVivo 11, a software package to facilitate the organization of qualitative analysis.

### Analysis

We used the themes from a synthesis of QES,  or *mega-ethnography*, as an a priori analytic framework.^1^ This mega-ethnography synthesized themes from 11 QESs^2,7–16^ and is the largest review of qualitative research on chronic pain. It synthesizes findings from 187 qualitative studies exploring the experiences of more than 5000 international participants with chronic pain. The a priori themes from this QES were the following: my life is impoverished and confined; I am struggling against my body to be me; I have lost my personal credibility; I am trying to keep up appearances; I need to be treated with dignity; I am on a quest for the diagnostic grail; deciding to end the quest for the grail is not easy. We felt that using an a priori research-based framework would allow us to build on existing research and focus on data that offered additional “dimensions, nuances, or insights (594).”^17^ In this way, we intended to combine a deductive and inductive approach to data analysis. This is the first study to use a priori themes from a review of QESs as an analytic framework for primary qualitative research.

Two researchers sorted the data using the a priori framework. If the data did not clearly fit the framework, we coded and sorted it into new themes through constant comparison. The aim of working collaboratively in this way was to develop ideas through discussion. We sent our coding report to a third researcher to verify our categories. Following analysis, we wrote a description of each theme, selected a verbatim narrative to exemplify this theme, and reworded the verbatim narrative to ensure confidentiality.

### Ethical Considerations

Internet-mediated research involves “the remote acquisition of data from or about human participants using the internet and its associated technologies.”^18^ Because the data involved comments posted online that were already in the public domain, we did not seek ethical permission to collect and analyze these posts. However, this decision was not straightforward. Our team discussions focused on the first two principles for human research stipulated in the *Code of Human Research Ethics*^19^: First, “respect for the autonomy, privacy and dignity.” Researchers should not consider research data to be *public* just because it is *accessible*.^18^ An underlying ethical principle is that you cannot carry out observational research, without participants’ consent, unless they are expecting to be observed.^19^ After discussion, we felt that YouTube viewers who posted comments would expect their comments to be observed. In 2013 when we put the film on YouTube, we had no intention to collate and analyze data arising from reactions to it. Rather, the data were “found texts” collected unobtrusively.^18^ We suggested that our research would not “pose additional threats to privacy.”^18^ However, the issue of public or private is a gray area. Internet researchers also need to be aware of the service agreements provided by the internet hosts: in this case, the YouTube terms of service stipulate that you cannot “collect or harvest any information that might identify a person.” A second principle of human research is “quality, integrity and contribution” of research.^19^ Geographical and temporal distance is a challenge for internet researchers. The researcher set at a distance has no way of controlling the research environment. For example, the researcher does not actually know whether the internet users are who they say they are. We approached the Medical Sciences Interdivisional Research Ethics Committee, University of Oxford, who were satisfied that we had followed the University of Oxford’s and British Psychological Society’s guidelines for internet-mediated research and that the data analysis did not require ethical approval. They provided a letter to that effect (R67327/RE001).

## Findings

We collected narrative data from all 323 posts on YouTube (15,986 words). We developed two inductive themes from data that explored the positive value and potential impact for viewers of watching the film and communicating via YouTube: (1) It has given voice to our suffering and (2) it makes me feel that I am not alone. Although the remaining data fitted the a priori framework, we identified two further subthemes that added “dimensions, nuances, or insights.”^17^ to the a priori framework: First, *I have had enough of me* added insight to the theme *my life is impoverished and confined*; second, *I am treated like a criminal because I take opioids* added insight to the theme *lost personal credibility*.

### It Has Given Voice to Our Suffering

This theme was developed from 106 YouTube posts. It describes the value of feeling “identified.” Forty-one viewers posted personal thanks for providing the film. Viewers describe the film as “speaking for us all” and giving voice to an invisible pain. The theme describes the value of having a *true* account of the chronic pain experience, which gave viewers a sense that they were finally heard.

Thank you for sharing this film. She is speaking for us all. I feel so identified. It’s like she’s talking about me. It’s like someone read my thoughts and feelings. You summed up my life in ten minutes. This is my life as it is happening right now. This film speaks so much about what I have been through over the last year. It is so hard not being understood and to struggle with pain every day: you have to “put on a mask” and smile because people don’t like to be around people who are suffering. Thank you for your honesty.

Viewers also described the film as a potential resource that could be used to explain to other people what it is like to live with chronic pain. They described how difficult it is to find the right words to describe pain so that people would understand.

Thank you so much for giving me the film to let people know how I feel. I have always struggled to explain how I feel and what I go through and somehow you have explained word for word what I go through. This film tells a story of all of us living with chronic pain and I am thankful it’s available for everyone to see. I now have something to share with people to help them understand my pain. I can now show my friends and family this film so that they can understand. It explains everything I found it hard to articulate. What a gift. I will share this film.

Some had shared the film with other internet viewers. Some felt that all health care professionals should watch and learn from it.

Thank you so much for this wonderful, educational film. The film may help someone on the edge. This is an important resource for people with chronic pain and those close to them. We need more health care professionals to watch this. Every doctor and policy maker should watch this. Thank you. Hopefully, if people become more aware of what it is like, people’s perception will change.

### It Makes Me Feel like I Am Not Alone

This theme was developed from 87 YouTube posts. It describes the value of feeling that you are not alone. Watching the film and commenting on it made viewers feel that there were people *out there* who understood and were in the same boat. This created a sense of community and support.

Thank you for posting this film and making me feel not alone. This is the first time I have seen anything that has acknowledged my pain. This gives me hope that there are people in the world who understand. It is such a sad existence, alone, trapped in your body: the film makes me feel like I’m not alone. It’s like everyone in pain shares my story. Only people in pain understand what it’s like. We have to stick together: stand strong for each other, you are not alone. We are in the same boat. I am here for you. We are here for each other. I know your pain.

### Criticism of the Film: She Doesn’t Look like She Is in Pain

There were seven posts that criticized the film. Five viewers did not think that the film portrayed a *true* experience and questioned the validity of the film. Two viewers would have preferred a *real* person in pain to play the role, rather than an actor. The essence of criticism was that the actor did not appear to be in pain:

This is not a true picture of someone in pain. She can drive. She can walk. She is carrying bags. She is making a cup of tea. I wouldn’t be able to ride the bus with my pain. How bad can her pain seriously be? It will make people think that pain is just in our heads.

However, other viewers challenged this criticism saying that even though pain is invisible, it is still real.

She may be just pushing herself through her pain and not showing it. I have pain but I have to carry my shopping: it just means that I can’t do anything else for the rest of the day. We are all trying our best not to just stay in bed. We need to move. Pain doesn’t stop your legs moving. You still need to keep going. No one is going to do everything for you. That’s a dangerous thing to say: it was pretty low.

### Deductive Analysis Using a Priori Themes

We found that the online data resonated with the a priori themes.

#### My Life Is Impoverished and Confined

The a priori theme described “the all-pervading nature of pain which invades all aspects of my day and night. Life is impoverished and confined. I am uncertain of what the future will bring and I am confined to live in the moment (5).”^1^ This theme is supported by viewers’ descriptions of life as “literally a living hell”:

Life is passing me by. To get through each day is a struggle. Pain has taken the place of joy. I can barely move. I feel like my body is shutting down. Pain is there day and night. Slowly dying and nobody can see it.

Viewers described a loss of social connection, exacerbated by feeling “useless,” “ashamed,” or “guilty” for not living up to expectations.

It feels like you’re alone. It’s a very lonely life. You lose your best friends, colleagues, and even your family. It makes me feel so sad when those close to you start to think that you are a constant drag. I feel rejected, misunderstood. I am not living up to people’s expectations. No one understands invisible pain. They don’t want to hear it. I have lost friends. I have lost relationships. No one gets it. They think that you can be cheerful all the time. I don’t belong. I have withdrawn from life.

##### Subtheme: I Have Had Enough of Me

The essence of this subtheme resonated with the a priori theme “my life is impoverished and confined.” However, we felt that it added an additional layer of pathos. It describes the feeling of not wanting to go on living in pain. Some felt that no one would care or miss them if they were gone. However, despite this feeling, viewers describe a strong will to keep living for those they love and emphasize the value of connection.

I have lost hope. I don’t think that things are going to get any better than this. This isn’t living. Sometimes I feel like ending it all. I’m never happy. I’ve been real close to calling it a day. Sometimes I pray that I don’t wake up. No one would miss me. I’m not important. No one would even notice if I wasn’t here. I feel ready to quit. I still have fight left in me. I still love this world and this life. I love my family. I still want to go on living. I live for my family. I live for my children who I love with all my heart. I am hanging on in this world for them.

#### I Am Struggling against My Body to Be Me

The a priori theme described “a struggle to maintain my sense of self. My body has become alien and malevolent and I cannot fulfil my normal duties. I am irreparably altered (6).”^1^ This theme was supported by viewers’ descriptions of pain as a robber or thief. Viewers paint a contrasting picture between their “old self” and the present self. This new fake self is “pathetic” and weak: the “sparkle” has gone.

Pain has changed me. I am losing my sense of self. Pain has robbed me of who I was. I have become bitter and twisted. It’s like carrying around an angry demon inside you that is trying to get out. Pain has killed the sparkle for life that I used to have. I have become a shadow of my old self. … I am no longer the happy, carefree, and energetic person that I once was. I used to be. Pain is killing me. It has stolen my life. I used to be the life and soul of the party. I miss the old me. I feel ashamed when once I was proud. I am nothing.

#### Lost Personal Credibility

The a priori theme described “loss of personal credibility. No one believes me because there is nothing to prove that my pain is real (7).”^1^ Similarly, viewers felt that other people thought that they were *faking*. They describe an “invisible illness” with no diagnostic proof. Viewers described this invisibility as the “worst part” of their chronic pain experience. They described a feeling of being set apart from society because of their invisible, stigmatized condition.

I know that people don’t believe me. This is worse than having cancer because you cannot see it. The doctors can’t measure it: there is no test to prove it. We have to fend for ourselves. Nobody believes that you are in pain. If only I could unzip my skin and show other people what it is like to live inside my body. Health professionals just say it is in your mind. People look down at me in disgust because they think that I am exaggerating. They think you are fine because there is nothing to show for it, like crutches or a wheelchair. I might look good on the outside but I am dying on the inside.

##### Subtheme: Treated like a Criminal because I Take Opioids

The essence of this subtheme resonated with the theme “lost personal credibility.” However, we felt that it added an additional layer that was specifically relevant to loss of credibility from opioid use. To reinforce their credibility, viewers drew a distinction between taking opioids for pain control and drug misuse, emphasizing that “I am not a drug addict.” This moral narrative was used to describe the narrator as a “good person”:

I *need* to take opioids; I don’t *want* to take them. I don’t take opioids to get high. My problem is pain, not addiction, but healthcare professionals treat me like my problem is addiction, not pain. They forget that I am in constant pain. Am I an addict just because I have to take pills? I don’t want to take them. I know that taking drugs can be your downfall: it can change your personality; it can damage your liver; it can affect other parts of your body. There has got to be another way to deal with pain.

Some felt that the stigma of taking opioids was heightened by legal restrictions on opioid prescription and that these restrictions were politically motivated, rather than motivated by efforts to improve patient care:

The government will not let health professionals treat us. … Even cancer victims have had their medication taken away. … Everyone is afraid of addiction and drug abuse. The government are bullying doctors who are afraid of losing their license. Health professionals are only trying to *help*, not *harm* people in pain. The government doesn’t care about the responsible, non-abusing people in pain.

#### Trying to Keep up Appearances

The a priori theme described the need to “put on a show and keep up appearances. I keep my pain to myself because I don’t want to be judged as being weak, and I don’t want to spoil things for everyone else. If I keep quiet about it no one will notice that I am no longer the person that I was (7).”^1^ Viewers describe the burden of keeping up appearances.

Don’t judge a book by the cover: looks can be deceiving. I keep it to myself. It is not what it seems. I don’t want people to think that I constantly complain. With an acute health problem people rally around and are there for you. Chronic pain just becomes normal and people don’t want to hear about it all the time. You have to smile and put on a mask. People don’t want to be around people who are sad and suffering. I don’t even tell my friends and family because I am afraid that I will end up alone. I just carried on in spite of pain and I didn’t complain: I will be in bed for a few days now. I wish that I could tell people what’s really going on. It’s hard to put on a brave face every day and act like you’re okay. I feel so fake.

#### I Need to Be Treated with Dignity

The a priori theme described “a negative experience of the healthcare system. No one is hearing my story or involving me in decisions about my care. I need to be treated with some dignity. I feel like a shuttlecock in the care system where nothing is being done to help. I feel like I am going around in circles (7).”^1^ Viewers describe the need to be treated with respect: to be treated as human beings with their own personal stories. They describe the need to be believed and not blamed for their condition.

It is frustrating to go to one doctor after the other. I have seen so many doctors that it feels like riding a misery-go-round. You end up feeling like you are not worthy of more tests and treatments. They need to treat people with respect: never make them feel that they are less. We’re in pain, not crazy. Please never write us off. We need doctors who believe what we are saying and not ones who rely solely on medical tests to judge what is real and what is not real. They need to listen and believe what we say. Who would go through all these humiliating appointments if there wasn’t anything wrong? I have been treated so poorly and I really need some compassion.

#### Quest for the Diagnostic Grail

The a priori theme described patients’ need for a medical diagnosis. “If the doctor can’t find anything then people will not believe me. I must have something or why would it hurt? I just want to find out what is wrong with me and so it can be cured.”^1^ Similarly, YouTube viewers described the search for a diagnosis and cure. Some were still holding onto hope for a “miracle” and would be willing to try anything to get rid of their pain:

I pray for a miracle cure. I tried every treatment I could find. I try and hold onto whatever sliver of hope that I can. I pray that a cure will come someday. I will keep searching for answers. I will keep looking for a cure even if there isn’t one. I hope that someday I will be able to wake up in the morning and reach for my dreams.

#### Deciding to End the Quest for the Grail Not Easy

The a priori theme described a person who is learning to live alongside his or her pain. This involved a realization that there is no fix and incorporated “the challenge of giving up the quest for a diagnosis and learning to live with pain (7).”^1^ Viewers described the need to accept that life is going to be different: you have to make the most of the positive moments. However, ending the quest for the diagnostic grail can be extremely challenging when you live in pain.

I’ve come to realise that my life has to be different. I have to create and to live a new *normal* that is acceptable to me and other people. We only have one life to live. Strive to improve every day and live life to the fullest. Focus your mind on things that bring you joy. Take the good moments. I focus on the things that make me happy. I will enjoy the days that my body lets me enjoy, and look after my body on bad days. It’s been a long journey to realise this but it has brought me some relief. Life is looking brighter. It can be difficult on tough days. It is hard to live life to the full when you are in pain and have to give up things that you love.

## Discussion

We aimed to find out what comments viewers made about the YouTube film and to explore potential value or impact through a qualitative analysis of online comments. Our findings indicate that watching the film and communicating online can provide a useful tool for expression, and viewers posted personal thanks for providing the film. We found that the film gave voice to people’s pain and made them feel that they were no longer alone. Pain defies language,^20,21^ and the need to give a voice to suffering holds particular relevance for those with long-standing pain. There is a large body of qualitative research showing that pain is experienced as illegitimate and, as such, is hidden from other people. The film was described as a tool that allowed people to feel as if they were “morally recognizable” to others (75).^22^ People in pain experience a tension between needing to give voice to pain and, at the same time, hiding pain.^1,2^ This tension contributes to the complexity of living with, and providing effective treatment for, chronic pain.

Internet spaces have become online communities where people share social interaction through virtual space,^23^ and the internet cannot be ignored as a source of knowledge for healthcare information and advice. Social media plays an integral role in how people share ideas and knowledge and also how people construct a sense of self and community.^24^ It is now an established route to health information and support.^25^ As many as 72% of adults in the United States search the internet to find out about health issues and 16% specifically search for other people with the same health issue.^26^ We found that the film was a stimulus for an internet community where a subgroup of people shared experiences, stories, and support about their pain. Internet users described feeling like they were no longer alone, that they were part of a community: this community is neither coherent, homogenous (nor even contemporaneous), but its sense of one-ness comes from shared invisible pain.

Internet-based research demonstrates that the simple process of posting a comment online can meaningfully create a sense of solidarity and support. We saw how viewers responded to the criticism that the actress “didn’t look like she was in pain” (“that’s a dangerous thing to say: it was pretty low”). Warwick and colleagues^27^ also found that internet “blogging” facilitated a virtual online support system: social support occurred across 100% of blogs, with bloggers and readers giving, seeking, and receiving support through their exchanges. Connecting through blogs appeared to provide comfort and reduce isolation. This resonated with our own findings where internet users posted stories about a lack of understanding and the need to hide their pain. Similarly, Tsai and colleagues found that blogging provided social support by allowing people to share their experience of chronic pain.^28^ The internet can provide an outlet for expression which allows users to reframe their experience,^29^ and it may be that the potential for positive impact lies in the production of narrative.^30^ We construct our sense of identity through stories.^31^ Charon^32^ and Frank^22,33^ advocate the importance of narrative in giving voice to suffering and allowing people to find meaning.

However, although our findings support the benefits of the online community for those in pain, there is also potential for harm. Ziebland and Wyke warn that “we cannot assume that the effects of exposure to online [personal experience] are always benign (240).”^25^ Malik and Coulson highlight some of these disadvantages in a study of internet support for infertility^34^; for example, reading about other people’s experiences (negative or positive), getting inaccurate information, unhelpful replies, or even experiencing hostility. There is the danger that information is unbalanced or conflicting. This can not only cause confusion and anxiety but also undermine “otherwise good, instinctive decision making (231).”^25^ Accessing stories on the internet can make people feel supported but also has the potential to lead to unrealistic positive or negative expectations. There is always the danger that the narrative is dominated or manipulated by a particular voice. It may also mean that people begin to rely on online relationships, rather than benefit from face-to-face social contact. Others have argued that there is a possibility that these accounts may not be true.^25^ Our findings did not reveal any evidence of harm, and this may be a limitation of using internet comments as data. Further research to explore negative experiences of watching the film would be useful.

The internet has created new opportunities but also challenges for research.^35^ Internet research ethics is defined as “the analysis of ethical issues and application of research ethics principles as they pertain to research conducted on and in the Internet.”^36^ There is currently no consensus on ethical guidelines for internet research, and it may be that research governance needs time to catch up with the digital revolution.^37^ Available guidelines suggest that consent, confidentiality, and anonymity are not required where the research is conducted in a public place where people would reasonably expect to be observed by strangers.^19^ However, the internet can blur the boundary between public and private space.^37^,^38^

Eysenbach and Till argue for informed consent^38^ and describe negative reactions from internet users whose words have been harvested without their knowledge or consent. As such, in a public internet blog, Zimmer discusses the ethics of harvesting data from public Twitter accounts.^39^ Ressler and colleagues^29^ found that 58% of bloggers did not share their blogs with their health care professional because they were concerned that they would be judged negatively; some also felt that being observed would hamper their freedom of expression. Ressler and colleagues offer some guidance for researchers to help them to determine the level of privacy that an internet user might expect: Did you have to subscribe or register to use the account? Are there tens or thousands of viewers? What are the stipulated norms or rules of the internet space (if any)?^29^ Some might argue that because we deconstructed narratives and rephrased the data if we felt that it would allow a reader to return to the source data we have sacrificed authenticity for the sake of confidentiality. Researchers making the choice to seek informed consent for the use of internet data need to consider both the practicalities of gaining consent and also the effect of this on the data. We suggest that when in doubt, one should seek ethical review.

Qualitative researchers using verbatim narrative need to consider what is “personally identifiable information” and the possible harms from bringing sensitive, embarrassing, or even illegal information to further scrutiny. At the outset (and after lengthy discussion) we regarded the space as public; however, as we began to analyze the data, this decision seemed less clear. For example, in developing the theme “I have had enough of me,” we began to consider the possible harms of being exposed: what might the effect be on family or friends? We chose to sacrifice the power of verbatim text to ensure confidentiality. Although this has added a layer of interpretation, we felt that it was a necessary safeguard.

For the first time, we used an a priori framework from a very large review of qualitative research exploring chronic pain, and this allowed us more time to focus our inductive analysis on new ideas and insights. Frameworks can usefully provide *sensitizing concepts* (“a general sense of reference and guidance [in] approaching empirical instances [7]”^40^) as long as researchers avoid the temptation to fit square pegs in round holes and take measures to challenge their categorizations. These posted comments come from a much larger body of viewers, and we only have the comments of those who chose to post them. Qualitative research aims to “make you think”^41^; it does not aim to generate statistically representative data. Therefore, a tally of how many codes make a theme does not add substance to the strength of a particular theme: like the story of the emperor’s new clothes, a single voice can speak the truth.

There are currently no agreed-upon criteria for evaluating the impact of qualitative research, and the impact of arts-based health research is rarely evaluated.^4,42^ Parsons and Boydell suggest that “impact might be a subtle shift in viewers’ perspectives (171).”^43^ In their evaluation of an arts installation to portray the experience of homelessness, Parsons et al. explore how the audience interacts with research findings portrayed in art.^44^ Lafrenière and Cox propose a Guiding Arts-Based Research Assessment framework^42^ for evaluating the impact of arts-based health research: namely,

(a) Does it generate emotions/feelings?

(b) Does it help the audience notice, understand, and appraise the issues?

(c) Does it generate internal dialogue or prompt interpersonal discussion so that it furthers engagement and response?

(d) Does it move the audience to change in salient ways?

Our findings indicate that the film generated emotions and feelings, that it encouraged understanding and appraisal, and that it generated dialogue and discussion. Our approach did not allow us to know whether or not watching and discussing the film online moved the audience to change in salient ways. Because the impact of qualitative research can be subtle, it remains a challenge to evaluate impact. Qualitative researchers should be mindful of impact from their research and consider innovative ways to explore its impact. Further research exploring the usefulness of the film in clinical settings would be helpful. For example, what would be the impact of asking patients with chronic pain to watch the film before a clinical consultation so that they might feel heard, understood, and not alone?

Our findings support the relevance of using the a priori framework for this group of internet users. Viewers’ stories described a life impoverished and confined by their pain, “struggling against my body to be me”; lost personal credibility; trying to keep up appearances; needing to be treated with dignity; the quest for the diagnostic grail; and the difficulty of giving up this quest. We found two themes that added a layer of pathos. First, my life is so profoundly impoverished that “I have had enough of me.” The original internet data powerfully described loss of hope, and yet for reasons of confidentiality we have not used these verbatim accounts. Second, “I am treated like a criminal for needing opioids” and this threatens my personal credibility. This data was generated during a time of legislative upheaval for opioid prescription in the context of an opioid epidemic.^45–48^ There is limited evidence for long-term opioid use for chronic pain outside of end-of-life care,^49–51^ and yet there has been a historic rise in opioid prescription^45–48^ and a parallel rise in death rate associated with opioid pain medication for chronic pain.^52,53^ In the United States, the  SUPPORT (Substance Use Disorder Prevention that Promotes Opioid Recovery and Treatment) for Patients and Communities Act recognises a need for tighter controls.^54^ Similarly, the Canadian government is taking action to address the opioid crisis.^55^ This issue is recognized by the World Health Organization.^56^ However, our findings suggest that some people in pain may see withdrawal of opioids as contributing to their already threatened personal credibility. Health interventions that focus on restoring a sense of personal credibility might be useful in the transition toward reduced opioid prescription.

## Conclusion

This is the first study to use a priori themes from a mega-ethnography of chronic pain as an analytic framework.^1^ In this way, we combined deductive and inductive approaches. We felt confident in using the framework because it was drawn from 11 QES reviews^2,7–16^ of 187 qualitative studies and more than 5000 people with chronic pain. Therefore, it was no surprise that the internet data resonated with the existing framework; we would have been surprised if it had not. This combined deductive and inductive approach allowed us to make best use of exiting research and focus on innovative data. We found it useful to use an a priori framework: this allowed us to build on existing research yet also identify additional “dimensions, nuances, or insights (594).”^17^ This is particularly important in view of the expanding qualitative research base. We have now identified more than 200 further primary qualitative research studies exploring the experience of chronic pain published since the mega-ethnography in 2012 and a synthesis of these further studies is currently under review. Future qualitative research should build on these foundations of knowledge and focus on specific areas that have not yet been explored. Qualitative researchers must seriously consider the implications of harvesting data from the internet and not assume that *easily accessible* is the same as *public*.
